# Black raspberry extract inhibits regulatory T-cell activity in a murine model of head and neck squamous cell carcinoma chemoprevention

**DOI:** 10.3389/fimmu.2022.932742

**Published:** 2022-08-09

**Authors:** Nathan M. Ryan, Felipe F. Lamenza, Puja Upadhaya, Hasan Pracha, Anna Springer, Michael Swingler, Arham Siddiqui, Steve Oghumu

**Affiliations:** ^1^ Department of Pathology, The Ohio State University Wexner Medical Center, Columbus, OH, United States; ^2^ Department of Microbiology, The Ohio State University, Columbus, OH, United States; ^3^ Department of Microbiology, Immunology, and Inflammation, Center of Neurovirology and Gene Editing, School of Medicine, Temple University, Philadelphia, PA, United States; ^4^ Kentucky College of Osteopathic Medicine, University of Pikeville, Pikeville, KY, United States

**Keywords:** black raspberries (*Rubus occidentalis*), HNSCC (head and neck squamous cell carcinoma), Treg - regulatory T cell, chemoprevention, T cell, immunomodulation

## Abstract

Head and neck squamous cell carcinomas (HNSCC) are one of the most diagnosed malignancies globally, with a 5-year survival rate of approximately 40% to 50%. Current therapies are limited to highly invasive surgery, aggressive radiation, and chemotherapies. Recent reports have demonstrated the potential phytochemical properties of black raspberries in inhibiting the progression of various cancers including HNSCCs. However, the effects of black raspberry extracts on immune cells of the tumor microenvironment, specifically regulatory T cells during HNSCC, have not been investigated. We used a mouse model of 4-nitroquinoline-1-oxide (4NQO) chemically induced HNSCC carcinogenesis to determine these effects. C57BL/6 mice were exposed to 4NQO for 16 weeks and regular water for 8 weeks. 4NQO-exposed mice were fed the AIN-76A control mouse diet or the AIN76 diet supplemented with black raspberry extract. At terminal sacrifice, tumor burdens and immune cell recruitment and activity were analyzed in the tumor microenvironment, draining lymph nodes, and spleens. Mice fed the BRB extract-supplemented diet displayed decreased tumor burden compared to mice provided the AIN-76A control diet. Black raspberry extract administration did not affect overall T-cell populations as well as Th1, Th2, or Th17 differentiation in spleens and tumor draining lymph nodes. However, dietary black raspberry extract administration inhibited regulatory T-cell recruitment to HNSCC tumor sites. This was associated with an increased cytotoxic immune response in the tumor microenvironment characterized by increased CD8^+^ T cells and enhanced Granzyme B production during BRB extract-mediated HNSCC chemoprevention. Interestingly, this enhanced CD8^+^ T-cell antitumoral response was localized at the tumor sites but not at spleens and draining lymph nodes. Furthermore, we found decreased levels of PD-L1 expression by myeloid populations in draining lymph nodes of black raspberry-administered carcinogen-induced mice. Taken together, our findings demonstrate that black raspberry extract inhibits regulatory T-cell recruitment and promotes cytotoxic CD8 T-cell activity at tumor sites during HNSCC chemoprevention. These results demonstrate the immunomodulatory potential of black raspberry extracts and support the use of black raspberry-derived phytochemicals as a complementary approach to HNSCC chemoprevention and treatment.

## Introduction

Head and neck squamous cell carcinoma (HNSCC) is the sixth most diagnosed malignancy globally and makes up approximately 4% of all cancers in the United States (1). It accounts for over 600,000 new cases and 350,000 deaths every year (2). The overall prognosis for patients is poor, with the 5-year survival rate for patients being 40%–50% (3). Primary risk factors for HNSCC development include tobacco and alcohol consumption, human papilloma virus (HPV16, 18), and exposure to environmental pollutants (4). Despite advances in research and new therapeutic approaches, improvements in patient outcomes have remained stagnant over the past three decades. Existing therapies are limited to disfiguring surgery, aggressive radiation, and chemotherapy (4). Even with successful resection, high rates of recurrence and metastasis further impede patient recovery (5). Given the poor prognosis and associated public health burden of HNSCC, current research is focused on novel chemopreventive and therapeutic approaches that improve patient survival as well as reduce tumor recurrence (5, 6).

Among the chemopreventive strategies against HNSCC, phytochemicals in black raspberries (BRBs) have shown promise in their anticancer effects (7, 8). In preclinical studies, BRBs have been shown to inhibit cancers of the head and neck, esophagus, and colon (9). Among the bioactive constituents contained in BRBs are anthocyanins, ellagitannins, and ellagic acid, which have antioxidative and anti-inflammatory properties (10–12). However, studies examining antitumoral properties of both BRB as well as individual metabolites have demonstrated that the complex mixture of bioactive constituents provide an advantage over individual compounds alone (13). While these individual BRB compounds are beneficial in delineating the mechanisms of cancer chemoprevention, BRB extracts that are enriched in the known bioactive compounds can enhance the efficacy of BRB-based cancer chemoprevention and provide additional insights into anticancer mechanisms of action. For example, BRB extracts and its phytochemical metabolites were demonstrated to show abrogation of the signal transducer and activator of transcription 3 (STAT3) phosphorylation mediated by IL-6 signaling (14). This is consistent with the established anti-inflammatory properties of BRB demonstrated by our group and others (10). Interestingly, a BRB extract but not its metabolites was capable of suppressing IL-2-induced STAT5 phosphorylation critical to the expansion of regulatory T cells (13). Although this study was performed *in vitro*, it demonstrates the potential of BRB extracts in the modulation of adaptive T-cell tumor immunity during cancer chemoprevention.

A major component of adaptive immunity to HNSCC is regulatory T cells (Tregs), which play an important role suppressing antitumor immune function (15, 16). Tregs are characterized by CD4^+^CD25^+^ expression and, though not unique to Tregs, by CD152 and GITR. FoxP3 is the primary nuclear transcription factor that regulates Treg differentiation and is induced through cellular interaction with the cytokines TGF-β and IL-2 (17). Induction of Tregs can promote carcinogenesis by suppressing immune responses through the release of inhibitory cytokines IL-10, IL-35, and TGF-β (18, 19). Once infiltrated into the tumor microenvironment, active Tregs overexpress inhibitory molecules CTLA-4, PD-1, TIM-3, LAG-3, and TIGIT, which together inhibit the antitumoral immune response (18–20). Combined, these cell surface receptors and the release of immunosuppressive factors mediate Treg immunosuppression of cytotoxic T lymphocyte, Th1, and antigen-presenting cell function (18, 19, 21). Given their important pro-tumoral role in HNSCC, Tregs present themselves as a potential target for HNSCC chemoprevention and therapy (15).

Previous *in vitro* studies on the impact of BRB extracts on IL-2-induced STAT5 phosphorylation (13) suggest a potential role for BRB extract-mediated regulation on Treg expansion and survival during HNSCC cancer chemoprevention. However, despite its currently understood success in modulating pro-inflammatory, apoptotic, and pro-survival markers, there is a significant lack of information regarding any potential impact of BRB extracts on Tregs during HNSCC chemoprevention using an appropriate *in vivo* model. Furthermore, a complete characterization of the effects of BRB extracts on immune cells of the tumor microenvironment during HNSCC *in vivo* has not been evaluated. This is crucial in order to fully determine the immunomodulatory role of BRB in HNSCC. In this study, we utilize the 4-nitroquinoline-1-oxide (4NQO) model of HNSCC to elucidate the impact of BRB immunomodulation on Tregs and other immune cells of the tumor microenvironment in HNSCC. This HNSCC model is commonly utilized to recapitulate early changes in head and neck squamous cell carcinoma oncogenesis in mice (22, 23). Studies using this model demonstrate an accumulation of Tregs in tumor microenvironments and is associated with disease progression during HNSCC as is found in HNSCC patients (24, 25). Our results demonstrate that BRB extracts modulate Treg accumulation in tumor microenvironments during HNSCC chemoprevention.

## Materials and methods

### Animal handling

C57Bl/6 mice (*n* = 30) aged 5 weeks were purchased from the Jackson Laboratory. Mice were equally distributed across sexes. All mice were kept on a 12-h day/night cycle and provided access to food and water *ad libitum*. Animal handling was kept in accordance with regulations maintained by University Laboratory Animal Resources. Animal protocols were approved by the Institutional Animal Care and Use Committee (Protocol #2018A00000054) and the Institutional Biosafety Committee of The Ohio State University.

### Animal diet

At week 0, mice were randomized into groups and placed on one of two diets beginning 3 weeks prior to 4NQO chemically induced carcinogenesis: standardized minimal nutrient rodent chow AIN-76A (4NQO, *n* = 10) or AIN-76A supplemented with black raspberry extract (BRB-E, *n* = 10). Additionally, one group (*n* = 10) received the standardized minimal nutrient chow AIN-76A without cancer induction by carcinogen exposure. Animals were maintained on their individual diets throughout the duration of the experiment. Once per week, food consumption was measured, and consumed amounts were replenished.

### Food and chemicals

BRB used in animal feed was purchased from the Stokes Berry Farm (Wilmington, OH, USA) before being shipped to Van Drunen Farms (Momence, IL, USA) for freeze drying. An ethanol BRB extract was generated as described in our previous study (26). Standard AIN-76A and its special formulations were prepared by Dyets Inc. (Bethlehem, PA, USA) and stored at −20°C until provided to the animals *ad libitum*. The carcinogen 4-nitroquinoline 1-oxide (4NQO) was purchased from Sigma-Aldrich (St. Louis, MO, USA; #N8141) and stored at room temperature away from light and humidity as directed by the manufacturer. Fresh 4NQO solution (100 µg/ml in drinking water) was prepared weekly and protected from light by foil-wrapped containers for the duration of administration to mice.

### Carcinogenesis

At week 3, mice were taken off in-line water supply. Water bottles were provided for mice designated for carcinogenesis containing 4NQO solution, at a volume of 300 ml per week. A sentinel group fed AIN-76A (*n* = 10) was maintained on regular drinking water at the same volume. 4NQO solution or water was replaced weekly. From week 16, all groups received clean drinking water without 4NQO supplementation. Water consumption and mouse weight were measured weekly. At week 24, mice were sacrificed. Tongue lesions and carcinoma counts were tallied as determined by gross inspection. Each tongue was visually inspected and tallied by three separate trained lab staff to ensure accuracy among all counts. Tongues were sectioned and preserved in 10% neutral buffered formalin followed by paraffin embedding, RNAlater, or snap freezing on dry ice.

### Flow cytometry

At terminal sacrifice, draining cervical lymph nodes and sections of the spleen were collected. Lymph nodes and spleens were passed through 70-µm nylon mesh to generate single-cell suspensions. Spleen suspensions were treated with ACK lysis buffer to ensure erythrocyte lysis. Cells were incubated with fluorochrome-conjugated antibodies: CD3-Alexa Fluor 700 (Cat# 100216), CD4-APC/Cyanine7 (Cat#100414), CD8-PerC/Cyanine5.5P (Cat# 100734), CD11b-FITC (Cat# 101206), CD86-APC/Cyanine7 (Cat# 105030), CD206-Alexa Fluor 700 (Cat# 141734), Ly6C-APC (Cat# 128016), Ly6G-PE (Cat# 127608), PD-1-Brilliant Violet 421 (Cat# 135218), PD-L1-PE Dazzle 594 (Cat# 124324), F4/80-Brilliant Violet 421 (Cat# 123132), and CD49b/DX5-FITC (Cat# 108906) (BioLegend, San Jose, CA, USA). Additionally, cells were intracellularly stained for Granzyme B, IFN-γ, interleukin (IL)-4, and IL-17. Prior to intracellular staining, cells were stimulated using cell activation cocktail (BioLegend, San Jose, CA, USA, #423303) containing phorbol-12-myristate 13-acetate (PMA) at 81 nM, ionomycin at 1.34 µM, and protein transport inhibitor (Brefeldin A) at 5 µg/ml for 6 h. Following stimulation, cells were intracellularly stained for Granzyme B-Brilliant Violet 711 (Cat# 506941), IFN-γ-APC (Cat# 505810), IL-4-PE/Cyanine7 (Cat# 504118), and IL-17-Brilliant Violet 711 (Cat# 506941) (BioLegend, San Jose, CA, USA). Samples were run using BD FACSAria III (BD Biosciences, San Jose, CA) with Red (640 nm), Yellow-Green (561 nm), UV (365 nm), and Blue (488 nm) lasers. Data analysis was conducted using FlowJo software (Tree Star Inc., Ashland, OR, USA).

### T-cell stimulation and ELISA

Spleen and lymph node single-cell suspensions were generated from their respective tissues. Cells were incubated in the presence of plate-bound αCD3 and soluble αCD28 antibodies (BioLegend, San Diego, CA, USA) for 72 h. The levels of the cytokines IFN-γ, IL-4, and IL-17 were analyzed from culture supernatants of stimulated and non-stimulated samples. Capture and detection antibodies for ELISA were purchased from BioLegend (San Diego, CA, USA).

### RT-qPCR

Tongue, lymph node, and spleen samples were stored in RNAlater (Thermo Fisher Scientific, Foster City, CA, USA) and frozen at −80°C for downstream analysis. Using TRIzol and a Bead Ruptor Elite (Omni International, Kennesaw, GA, USA) the tissues were lysed and homogenized. RNA from the tissues was extracted using the Direct-zol RNA Miniprep kit according to the manufacturer’s protocol (Zymo Research, Irvine, CA, USA) and reverse transcribed to cDNA using the High-Capacity cDNA Reverse Transcription Kit (Applied Biosystems, Foster City, CA, USA). Primer sequences for PCR were created using the IDT RealTime qPCR Tool (https://www.idtdna.com/scitools/Applications/RealTimePCR/, Integrated DNA Technologies, Coralville, IA, USA) (22, 27) Real-time PCR of cDNA samples was performed using the PowerUp SYBR Green Master Mix (Thermo Fisher Scientific, Foster City, CA, USA) with beta actin (*Actb*) as a reference gene. Gene transcripts amplified include *Foxp3*, *Il2r*, and *Ctla4*.

### Histology

Tongues, lymph nodes and spleens were fixed in 10% neutral buffered formalin followed by paraffin embedding. Paraffinized tissue sections 5 μm thick were cut for histopathology and immunofluorescence staining. Samples were stained with hematoxylin and eosin. For immunofluorescence, sections were rehydrated in xylenes and ethanol before heat-induced epitope retrieval using sodium citrate antigen retrieval buffer. Sections were blocked for 30 min in 10% normal goat serum before overnight incubation with rat anti-mouse CD4 or CD8 (Invitrogen, 14-0042-82; 14-0808-82) and rabbit anti-mouse FoxP3 or Granzyme B (Cell Signaling, 12653S; Abcam, ab4059). The following day, samples were incubated for 1 h with goat anti-rabbit H&L AF488-conjugated antibody or goat anti-rat H&L AF555-conjugated antibodies (Invitrogen, A11034; A21434). Following secondary antibody staining, each tissue section was then counterstained with DAPI (BioLegend, San Diego, CA). Confocal imaging was performed using a Zeiss LSM 700 confocal microscope (Carl Zeiss, Munich, Germany) and analyzed with the FIJI package in ImageJ.

### Western blot

Western blotting on tongue tissue lysates was performed according to standard procedures. Briefly, 40 µg of the total extracted proteins was loaded on 10% resolving gel and transferred onto 0.2-µm pore size PVDF membranes. Blots were blocked in 5% skim milk for 1 h and incubated with FoxP3 rat anti-mouse (14-5773-82, eBioscience) and GAPDH rabbit anti-mouse (2118S, Cell Signaling Technology) primary antibodies overnight. Blots were then incubated with goat anti-rat IgG HRP-linked secondary antibody (31470, Thermo Fisher Scientific, Rockford, IL) for FoxP3 and goat anti-rabbit IgG HRP-linked secondary antibody (31460, Thermo Fisher Scientific, Rockford, IL) for GAPDH. Chemiluminescence was detected by ECL Western blotting substrate (ThermoScientific, Waltham, MA) using the UVP Chemstudio imaging system (Analytik Jena, Beverly, MA).

### Statistical analysis

Analysis was performed using GraphPad Prism v8.0.2 (GraphPad Software, San Diego, CA, USA). Student’s *t*-test was used to determine statistically significant differences. Comparisons for significance were performed between sentinel and 4NQO groups as well as between 4NQO and 4NQO + BRB-E groups. Normality of data was verified by the Shapiro–Wilk test. Statistical significance was determined by a *p*-value threshold of 0.05.

## Results

### Black raspberry extract inhibits HNSCC carcinogenesis

**Figure 1:**
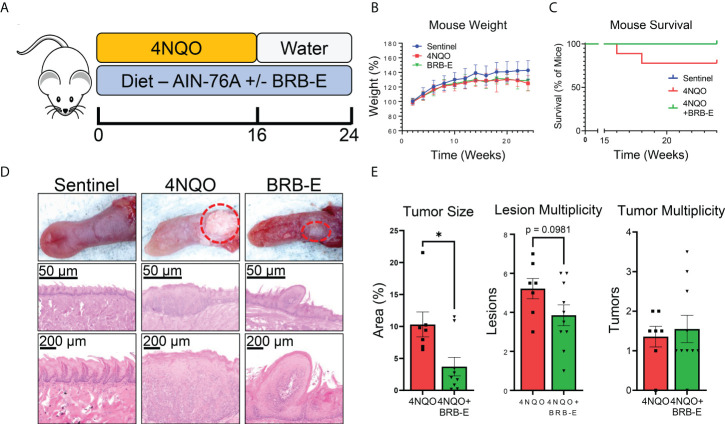
Black raspberry extract (BRB-E) inhibits HNSCC carcinogenesis in a carcinogenesis model of head and neck squamous cell carcinoma. **(A)** Study design of the 4NQO carcinogen-induced murine model of HNSCC and dietary BRB-E chemoprevention. **(B)** Percent weights of mice over time throughout the duration of the HNSCC cancer chemoprevention study. Data are represented as the percent weight over time relative to the initial weight of the mice. **(C)** Kaplan–Meier survival probability of non-carcinogen-induced mice (sentinel), and carcinogen-induced mice fed the control AIN76 diet (4NQO) or the BRB-E-supplemented diet (4NQO+BRB-E). **(D)** Representative images of tongues taken from mice in each group and images of representative H&E-stained tongues from each group at 50× and 200× total magnification. **(E)** Total percent of tongue surface area bearing tumors determined by image analysis. Total lesion and tumor count on each tongue taken from mice at terminal sacrifice. Each point represents the average of counts taken by three researchers trained to identify lesions or tumors taken in a blinded manner. *p-value < 0.05; for comparisons of BRB-E treatment groups to 4NQO-exposed control diet group using Student’s *t*-test. Sentinel (*n* = 10), 4NQO (*n* = 7), and BRB-E (*n* = 10).

Mice were provided with diets consisting of either control feed AIN-76A or AIN-76A diet supplemented with BRB extract (BRB-E). Experimental groups were administered with the carcinogen 4NQO in their drinking water. We analyzed the impact of the BRB-E-supplemented diet on HNSCC using the 4NQO-induced carcinogenesis model (**Figure 1A**). Mouse weights were monitored for the duration of the experiment, and we observed no differences in overall weight change between the carcinogen-induced mice fed the control diet and carcinogen-induced mice receiving the BRB-E-supplemented diet by week 24 (**Figure 1B**). Our analysis of overall survival rates showed that carcinogen-induced mice receiving the AIN-76A control diet had a 23% reduction in survival at week 24 compared to carcinogen-induced mice receiving the BRB-E-supplemented diet, which had no deaths at 24 weeks post-4NQO administration (**Figure 1C**). At terminal sacrifice, tongues were harvested for gross and histological analysis of tumors and lesions. Overall tumor sizes in carcinogen-induced mice fed the AIN-76A control diet were consistently larger than tumors on carcinogen-induced mice fed the BRB-E-supplemented diet (**Figure 1D**). Image analysis of tongue tumors demonstrated that tumors represent a statistically significantly larger percent of the total tongue surface area in carcinogen-induced mice fed the control AIN-76A diet compared to the BRB-E-supplemented diet (**Figure 1E**). Histological analysis of the tongues has revealed a reduction in hyperplastic and dysplastic lesions, and invasive capacity in mice fed the BRB-E-supplemented diet compared to the AIN-76A control diet. There was also a slight reduction in lesion multiplicity, but not tumor multiplicity in 4NQO-induced mice fed the BRB-E compared to the AIN-76A control diet (**Figure 1E**). These results corroborate previous findings on the anticancer effects of the whole BRB powder-supplemented diet on oral carcinogenesis in a 4NQO rat model (27). Taken together, our data demonstrate that dietary administration of an ethanol BRB extract limits tumor development, decreases overall tumor burden, and promotes overall survival in a mouse HNSCC model.

### Effect of black raspberry extract on CD4 T-cell differentiation during HSNCC chemoprevention

**Figure 2:**
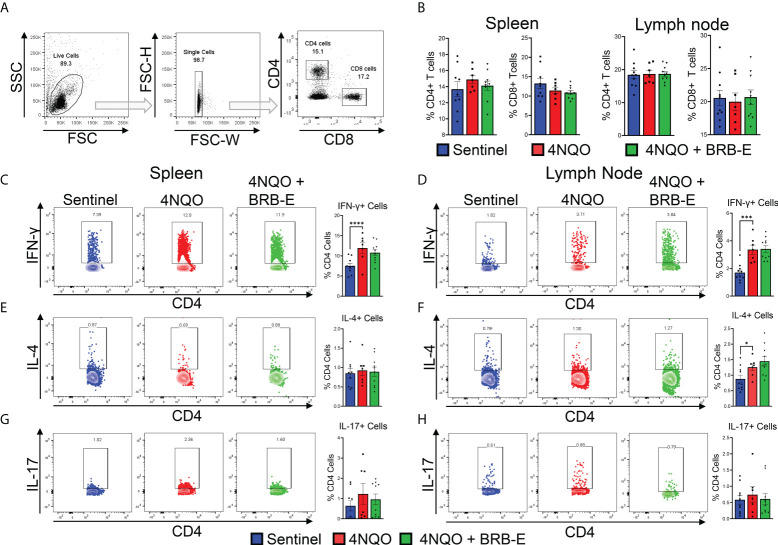
Effect of black raspberry extract on CD4 T-cell differentiation during HSNCC chemoprevention. **(A)** Gating strategy utilized to identify CD4^+^ and CD8^+^ T cells from the draining cervical lymph nodes or spleens of HNSCC tumor-bearing or sentinel mice by flow cytometry. **(B)** Proportions of CD4+ and CD8+ T cells in spleens and lymph nodes of non-carcinogen-induced mice (sentinel) and carcinogen-induced mice fed the control AIN76 diet (4NQO) or the BRB-E-supplemented diet (4NQO+BRB-E). **(C–H)** CD4^+^ T cells were examined by flow cytometric analysis for the presence of intracellular IFN-γ (C and **(D)**, IL-4 **(E, F)**, or IL-17 **(G, H)** in spleens **(C, E, G)** and lymph nodes **(D, F, H)** of mouse groups. Flow plots show a representative sample of the individual groups. Legend is applicable for B–H. *p-value < 0.05;; ***p-value < 0.001; ****p-value < 0.0001 for comparisons of BRB-E treatment groups to 4NQO-exposed control diet group using Student’s *t*-test.

Our analysis of the transcriptome of tongue RNA isolated from 4NQO**-**exposed and 4NQO + BRB-E-treated mice suggested to us the possible immunomodulation of T-cell populations driving the observed antitumoral impact of BRB-E treatment. As such, we examined overall CD4^+^ or CD8^+^ T-cell populations within both the spleen and the draining cervical lymph nodes of the sentinel, 4NQO, and 4NQO + BRB-E groups (**Figure 2A**). Our flow cytometric analysis of the total T-cell populations revealed no significant difference in CD4^+^ or CD8^+^ T-cell populations between carcinogen-induced and non-carcinogen-induced mice, or between carcinogen-induced mice fed the control AIN-76 diet or the BRB-E**-**supplemented diet (**Figure 2B**). Next, we determined the impact of carcinogen induction and BRB-E administration on Th1, Th2, and Th17 cell differentiation in draining lymph nodes and spleens of mice. To do this, we performed flow cytometric analysis of intracellular markers associated with each helper T-cell subset after *ex vivo* restimulation with PMA/Ionomycin with Golgi stop. In the spleens and lymph nodes, we observed a significant increase in IFN-γ^+^ CD4 T cells in carcinogen-induced mice compared to non-carcinogen-induced mice, a common antitumor response during experimental HNSCC in immunocompetent mice (**Figures 2C,D**). Interestingly, BRB-E administration did not inhibit Th1 differentiation in the spleens and lymph nodes of carcinogen-induced mice, as demonstrated by similar levels of IFN-γ^+^ CD4 T cells between 4NQO- and 4NQO + BRB-E-treated mice (**Figures 2C,D**). No differences were observed in IL-4^+^ or IL-17^+^ CD4 T cells in the spleens of carcinogen-induced and non-carcinogen-induced mouse groups (**Figures 2E,G**). In the draining lymph nodes, slight increases in CD4^+^ IL-4^+^ T cells were observed in both carcinogen-induced mouse groups fed the control AIN-76 diet and the BRB-E**-**supplemented diet compared to non-carcinogen-induced mice (**Figure 2D**). However, BRB-E administration did not affect IL-4 and IL-17 cytokine-producing CD4 T cells. Taken together, our results demonstrate that dietary BRB-E administration does not affect Th1, Th2, and Th17 differentiation in primary lymphoid organs in 4NQO carcinogen-induced experimental HNSCC.

### Regulatory T-cell recruitment to HNSCC tumor sites is inhibited by dietary black raspberry extract administration

**Figure 3:**
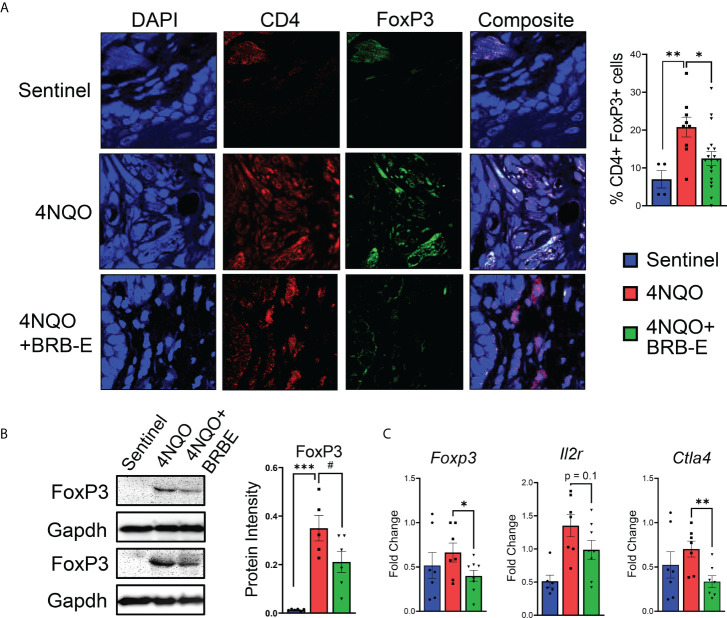
Regulatory T-cell recruitment to HNSCC tumor sites is inhibited by dietary black raspberry extract administration. **(A)** Multiplex confocal microscopy images of representative tongues taken from each mouse group. For each image, individual stains and composite images are shown. Each tongue was stained for DAPI (blue), CD4 (red), and FoxP3 (green). FoxP3^+^ CD4^+^ cells were quantified using ImageJ. **(B)** Western blot demonstrating FoxP3, and GAPDH protein content of tongue lysates isolated from sentinel, 4NQO, and 4NQO+BRB-E groups. Average protein intensity of Western blot bands was measured for several samples using ImageJ. **(C)** RT-qPCR data of tongue homogenate taken from sentinel or cancer-bearing mice receiving the control diet or the black raspberry extract supplemented feed for *Foxp3, Il2r*, and *Ctla4*. Data are represented as fold change relative to the *Bactin* control. *p-value < 0.05; **p-value < 0.01; ***p-value < 0.001; #*p*-value < 0.1 for comparisons of BRB-E treatment groups to the 4NQO-exposed control diet group using Student’s *t*-test.

Prior studies suggest that BRB extract can potentially impact T reg development *in vitro* (13). Given the pro-tumoral role of Tregs in HNSCC, we determined the impact of dietary BRB-E administration on Tregs during HNSCC chemoprevention *in vivo* using our experimental model. Immunofluorescence staining of tongue tissues taken from carcinogen-induced and non-carcinogen-induced mouse revealed an increase in CD4^+^ FoxP3^+^ T cell accumulation in the tumors of carcinogen-induced mice compared to non-carcinogen-induced control mice (**Figure 3A**). Interestingly, we observed a significant reduction in tumor-infiltrating CD4^+^ FoxP3^+^ T cells in 4NQO-exposed mice receiving BRB-E-supplemented feed compared to those receiving only the AIN-76 control diet. These data suggest that reduction in Tregs is a potential mechanism underlying BRB-E-mediated HNSCC chemoprevention. Further analysis revealed significantly decreased *Foxp3* gene and protein expression in tongue lysates of carcinogen-induced mice fed the BRB-E diet compared to carcinogen-induced mice fed the AIN-76 control diet (**Figures 3B,C**), as determined by RT-qPCR and Western blot, respectively. We next examined the expression of *Il2r* and *Ctla4*, surrogate markers of regulatory T cells, in tongue lysates of all mice experimental groups. We observed reduced expression of *Ctla4* in carcinogen-induced mice administered the BRB-E diet compared to mice fed the control diet, suggesting a reduction in Treg activity in the tumor microenvironment of carcinogen-induced mice fed the BRB-E diet (**Figure 3C**). Taken together, our data demonstrates that BRB-E-mediated chemoprevention is associated with a reduction in Treg recruitment to the tumor microenvironments in HNSCC.

### Black raspberry extract enhances antitumoral CD8 effector T-cell activity in the HNSCC tumor microenvironment

**Figure 4:**
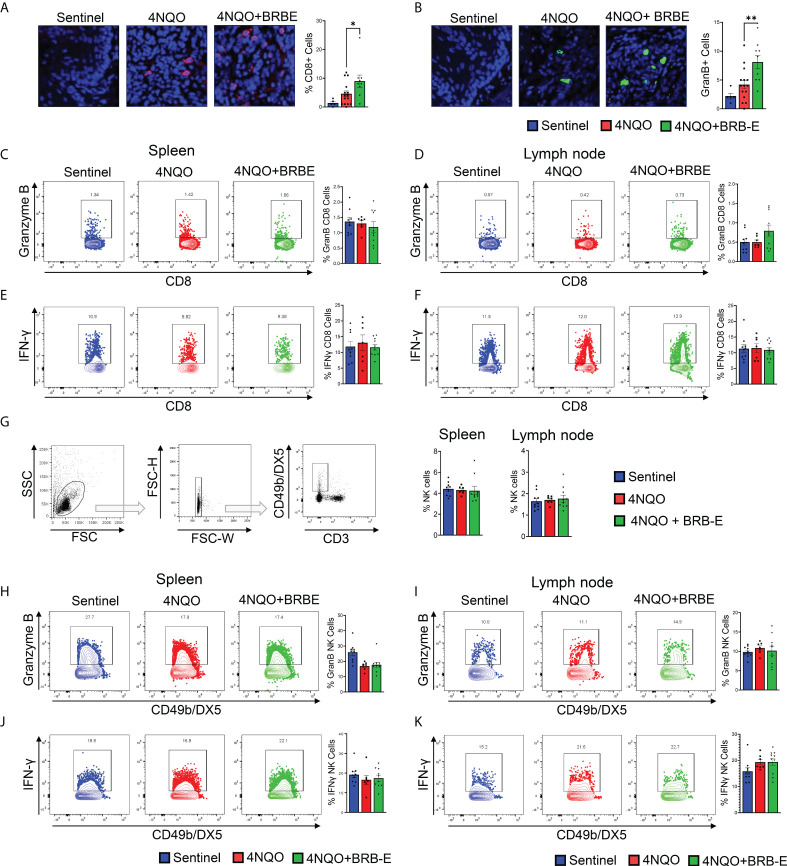
Black raspberry extract enhances antitumoral CD8 effector T-cell activity in the HNSCC tumor microenvironment. **(A)** Representative immunofluorescent images of tongue sections showing tumor-infiltrating CD8^+^ cells (red) in non-carcinogen-induced mice (sentinel) and carcinogen-induced mice fed the control AIN76 diet (4NQO) or the BRB-E-supplemented diet (4NQO+BRB-E). Tissues were counterstained with DAPI (blue) and quantified using ImageJ. **(B)** Representative immunofluorescent images of Granzyme B^+^ cells (green) in tongue sections of non-carcinogen-induced mice (sentinel) and carcinogen-induced mice fed the control AIN76 diet (4NQO) or the BRB-E-supplemented diet (4NQO+BRB-E). Tissues were counterstained with DAPI (blue) and analyzed using ImageJ. (**C** and **D**) Representative flow cytometric plots and population distributions of CD8^+^ Granzyme B^+^ cells in the spleens **(C)** and lymph nodes **(D)** of control feed or berry-treated tumor-bearing mice. Data are presented as percentage of total CD8^+^ T cells. (E and F) Representative flow cytometric plots and population distributions of CD8^+^ IFN-γ^+^ cells in the spleens **(E)** and lymph nodes **(F)** of control feed or berry-treated tumor-bearing mice. Data are presented as percentage of total CD8^+^ T cells. **(G)** Gating strategy for identification of NK cells from the draining cervical lymph nodes or spleens of HNSCC tumor-bearing or sentinel mice by flow cytometry. Proportions of NK cells in spleens and lymph nodes of non-carcinogen-induced mice (sentinel) and carcinogen-induced mice fed the control AIN76 diet (4NQO) or the BRB-E-supplemented diet (4NQO+BRB-E) are shown as bar graphs. **(H–K)** Representative flow plots demonstrating Granzyme B-positive staining of CD49b/DX5^+^ natural killer cells, isolated from spleens **(H)** and lymph nodes **(I)** of non-carcinogen-induced mice (sentinel) and carcinogen-induced mice fed the control AIN76 diet (4NQO) or the BRB-E-supplemented diet (4NQO+BRB-E). Representative flow plots demonstrating CD49b/DX5^+^ IFN-γ ^+^ natural killer cells isolated from spleens **(J)** and lymph nodes **(K)** of non-carcinogen-induced mice (sentinel) and carcinogen-induced mice fed the control AIN76 diet (4NQO) or the BRB-E-supplemented diet (4NQO+BRB-E). *p-value < 0.05; **p-value < 0.01 for comparisons of BRB-E treatment groups to the 4NQO-exposed control diet group using Student’s *t*-test.

Our observation of reduced Treg accumulation at tumor sites of BRB-E-fed carcinogen-induced mice led us to explore the potential impact of these cell populations on the antitumoral immune response within the HNSCC tumor microenvironment. Tregs mediate their activity in part by inhibiting the activity of effector antitumoral cytotoxic T cells. Interestingly, we observed that BRB extract administration increased the number of tumor-infiltrating CD8^+^ T cells in the tumor microenvironment (**Figure 4A**). To determine antitumor effector activity, we analyzed Granzyme B production. We also observed increased Granzyme B expression in the tumor microenvironment of BRB-E-administered tumor-bearing mice, indicating greater potential cytotoxic activity following BRB-E administration (**Figure 4B**). These data are supportive of and may provide a potential mechanism underlying our observation of the reduced tumor sizes in BRB-E-administered, carcinogen-induced mice compared to carcinogen-induced mice fed the control AIN-76 diet. Since we observed BRB-E-mediated modulation of cytotoxic T cells in the tumor microenvironment, we next explored Granzyme B production among CD8+ T cells within the spleen and draining lymph nodes of these mice. No significant differences in Granzyme B production were observed, suggesting that this activity was restricted to the tumor microenvironment (**Figures 4C,D**). To determine if other effector functions were impacted systemically by BRB-E diet, we examined IFN-γ^+^ production by CD8+ T cells in the spleen and draining lymph nodes of these mice. Similar to Granzyme B, BRB-E administration did not affect IFN-γ production by CD8^+^ T cells in the spleen and draining lymph nodes of carcinogen-induced mice (**Figures 4E,F**). Given that NK cells are an important component of immunity to HNSCC, similar analysis of Granzyme B and IFN-γ production by NK cells was performed. Similar to CD8^+^ T cells, BRB-E administration did not affect Granzyme B (**Figures 4G,H**) and IFN-γ (**Figures 4I,J**) production by NK cells in the spleens and lymph nodes of carcinogen-induced mice. Taken together with our previous data, these results demonstrate that the reduction of Tregs is associated with increased CD8 T-cell cytotoxic activity in the tumor microenvironment during HNSCC chemoprevention by dietary BRB-E administration.

### Impact of black raspberry extract on myeloid cell populations involved in immune response to HNSCC

**Figure 5:**
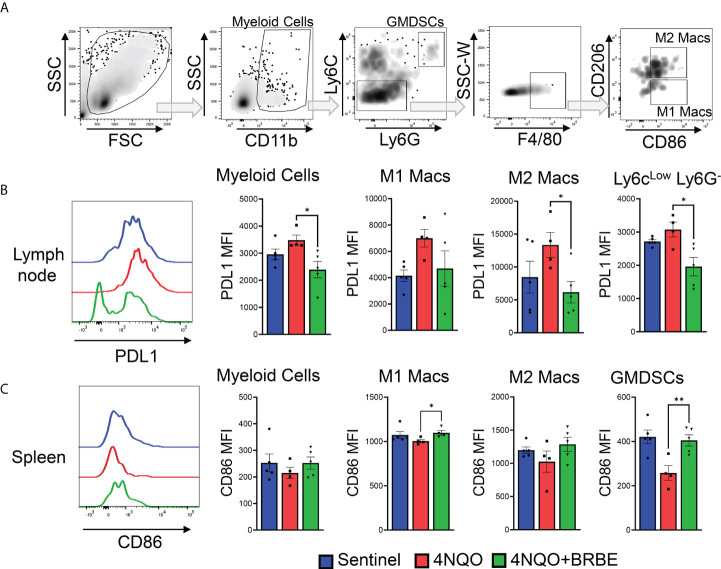
Impact of black raspberry extract on myeloid cell populations involved in immune response to HNSCC. **(A)** Flow cytometric gating strategy used to define CD11b^+^, CD11b^+^Ly6G^-^Ly6C^-^, CD11b^+^Ly6G^+^Ly6C^hi^, F4/80^+^CD206^-^, and F4/80^+^CD206^+^ cell populations within all analyzed tissues. **(B)** Representative flow cytometric histogram plots of PD-L1^+^CD11b^+^ cells. Bar graphs of PD-L1 expression cells on myeloid cells, M1 and M2 macrophages, and Ly6c^low^ Lg6G^-^ myeloid cell populations in the lymph nodes of non-carcinogen-induced mice (sentinel), and carcinogen-induced mice fed the control AIN76 diet (4NQO) or the BRB-E-supplemented diet (4NQO+BRB-E). Data are presented as mean fluorescence intensity (MFI). **(C)** Representative flow cytometric histogram plot of CD86^+^ M1 macrophage population in the spleens of each mouse group. Bar graphs of CD86^+^ cells from myeloid cells, M1 and M2 macrophages, and GMDSC cell populations in the spleens of non-carcinogen-induced mice (sentinel), and carcinogen-induced mice fed the control AIN76 diet (4NQO) or the BRB-E-supplemented diet (4NQO+BRB-E). Data are presented as mean fluorescence intensity (MFI). *p-value < 0.05; **p-value < 0.01 for comparisons of BRB-E treatment groups to the 4NQO-exposed control diet group using Student’s *t*-test.

In order to completely characterize the effects of BRB-E on immune cells involved in immune responses to HNSCC, we investigated the effects on the myeloid cell populations, and key costimulatory and immunosuppressive markers. Previous studies by our group demonstrated that dietary BRB administration reduced the expression of proinflammatory biomarkers of HNSCC, including *cxcl1*, *Mif*, and *Nfe212*, which are crucial to the development of immunosuppressive myeloid cell populations (10). We therefore analyzed these myeloid cell populations, as well as mediators of their immunosuppressive (pdl1) and costimulatory (cd86) activity by flow cytometry (**Figure 5A**). Our analysis of myeloid cell populations within the tumor draining lymph node showed an overall decrease in the expression of the co-inhibitory marker PD-L1 in myeloid cells, in carcinogen-induced mice fed the BRB-E diet compared to carcinogen-induced mice fed the control AIN-76 diet. This was especially noticeable in the M2 macrophage and Ly6c^low^ Ly6G^-^ sub-populations (**Figure 5B**). In the spleen, we observed a greater increase in the expression of the co-stimulatory marker CD86 in M1 macrophages and GMDSC cell populations within the BRB-E-treated carcinogen-induced mice (**Figure 5C**). These results may suggest that the reduction in proinflammatory mediators by BRB during HNSCC chemoprevention is associated with inhibition of immunosuppressive pathways in myeloid cells.

## Discussion

This study defines a novel mechanism of BRB phytochemical-mediated chemoprevention of tumor development and growth in HNSCC. We demonstrate that a diet supplemented with an ethanol extract BRB significantly inhibits the development of dysplastic lesions and progression of carcinoma *in situ* and reduces mortality caused by HNSCC. Furthermore, we provide evidence that this effect is associated with a reduction in regulatory T-cell recruitment and activity at oral tumor microenvironments and increased antitumoral cytotoxic CD8^+^ T-cell activity, characterized by Granzyme B production. Interestingly, our results indicate that the majority of BRB-E-mediated effects on T-cell immune responses are localized at the site of the primary tumor, suggesting an effect resulting from the topical delivery of bioactive phytochemicals. Furthermore, our study exemplifies the potential of BRB and its complex phytochemical constituents as potential chemopreventive agents with the ability to modulate T cells involved in immunity against HNSCC.

Due to its well-documented ability to recapitulate human HNSCC, the murine 4NQO model is routinely utilized in chemoprevention studies of the oral cavity (22, 23, 28). Although tumors that arise from this model make extensive functional characterization of immune cell populations in the tumor microenvironment technically challenging, it remains one of the best models that mimic the stepwise progression observed in HNSCC patients. Using these and other related animal models, we and others have demonstrated the chemopreventive potential of BRB in HNSCC chemoprevention (10, 27, 29, 30). While multiple mechanisms have been proposed to mediate HNSCC chemoprevention, including modulation of tumor suppressor and apoptotic genes, as well as proinflammatory, glycolytic, and glucocorticoid signaling pathways (27, 29–31), modulation of adaptive immune cells in a manner that promotes tumor cell cytotoxicity in the tumor microenvironment is a novel and potentially major mechanism of HNSCC chemoprevention by BRB. This is evident by the fact that although tumor multiplicity was unchanged due to BRB-E treatment in our study, tumor growth was significantly reduced by BRB-E administration. Furthermore, BRB-E administration reduced mortality in carcinogen-induced mice, indicating the efficacy of this BRB chemopreventive strategy in preventing tumor progression at later stages of development, where antitumor immunity is crucial. These immunomodulatory properties of phytochemicals in BRB support the development of these class of agents as complementary agents in the management of HNSCC (32).

While the antitumoral properties of BRB have been demonstrated by us and others in use against cancers including head and neck (27, 33, 34), esophageal (35), colorectal (36), cervical (37), and skin (38), the precise cellular mediators of BRB-mediated chemoprevention have yet to be fully understood. A previous study in a murine skin cancer model showed a correlation between topical exposure to BRB and a decrease in the infiltration of Treg to the primary tumor site (38). Given that increased Treg infiltration within the tumor microenvironment in HNSCC tumor-bearing patients has been more frequently associated with a worse patient prognosis (39, 40), understanding the impact of dietary BRB-E on Tregs is crucial to advancing our knowledge on BRB phytochemical anti-HNSCC mechanism of action. Our results demonstrate that dietary BRB-E significantly inhibits CD4^+^ FoxP3^+^ Treg accumulation within the primary tumors of the oral cavity, as evidenced by a reduction in Treg numbers as well as by decreased mRNA transcripts in the tongue for *Foxp3* and *Ctla4*, markers indicative of Treg recruitment and activity (18). Mechanisms that mediate this reduced recruitment of Tregs to HNSCC tumor microenvironments following BRB-E administration are still under active investigation, although chemokine-dependent infiltration *via* Ccl1, Ccl18, and Ccl22, and Vegfa-dependent recruitment are potential mechanisms (41). Indeed, BRB has been shown to inhibit angiogenesis in oral and esophageal cancers (42, 43).

BRB-E-mediated reduction of Tregs in HNSCC carcinogen-induced mice was associated with an increase in CD8^+^ tumor-infiltrating cytotoxic T lymphocytes in the tumor microenvironment. CD8+ T-cell infiltration is associated with improved prognosis in HNSCC (44). Increased CD8^+^ T-cell activation marked by heightened levels of Granzyme B secretion have been observed to have an antitumoral effect *in vivo* (45), and is associated with a positive prognosis in HNSCC (46). Our data demonstrates another potential immunomodulatory mechanism of BRB-mediated chemoprevention of HNSCC. We are investigating whether this is a direct effect of BRB-E or it is mediated by effects on Treg cells in the tumor microenvironment. Interestingly, we observed that the effects of BRB-E on Granzyme B production by CD8+ T cells were restricted to the primary tumor site, suggesting a potential link to BRB effects on CD4^+^ FoxP3^+^ Tregs in the tumor microenvironment. Future studies will further clarify this relationship between CD4^+^ FoxP3^+^ Tregs and cytotoxic CD8 T cells in the HNSCC tumor microenvironment following BRB-E administration. More in-depth functional analysis of antitumoral CD8+ T-cell activity using sorted CD8 T cells from carcinogen-induced mice fed the normal diet or the BRB-E-supplemented diet, to determine the molecular profile of tumor-associated CD8+ T cells as well as their ability to promote tumor cell lysis *ex vivo*, will be required to validate these effects. The current study, which defines the immune landscape of the tumor microenvironment during BRB-associated HNSCC chemoprevention, provides a strong rationale and basis for these future studies. Taken together, we demonstrate that BRB-E promotes an antitumoral immune landscape consisting of reduced Tregs and associated increase in antitumoral CD8^+^ T cells with Granzyme B production resulting in reduced tumor burden.

Beyond cells of the lymphoid lineage, Tregs are also known to have direct and indirect involvement with myeloid cell populations. Through CTLA4-dependent interactions with CD11b^+^ antigen-presenting cells, Tregs may inhibit the expression of the costimulatory molecules CD80 and CD86, necessary for appropriate antigen presentation (47). In our study, we found both decreased *Ctla4* transcripts in the tongues taken from mice administered with BRB-E and a concomitant increase in CD86 expression on myeloid cells globally in BRB-E-fed mice. Together, this demonstrates that BRB-E enhances the antitumoral properties of both myeloid and lymphoid immune populations, and potentially reduces Treg and myeloid cellular cross-talk necessary to promote an immunosuppressive tumor microenvironment. PDL1 may also mediate enhanced suppression by Tregs and promote Treg differentiation within the primary tumor (48). Our data support this finding, indicating that reduced FoxP3^+^ Tregs associated with a reduction in PD-L1 expression by myeloid cells in tumor-bearing mice administered a BRB-E-supplemented diet.

In conclusion, using the 4NQO carcinogen-induced model of HNSCC, we demonstrate that dietary BRB-E reduces tumor burden and incidence of pre-cancerous lesions, resulting in decreased mortality. BRB-E administration in carcinogen-induced mice attenuates the differentiation and infiltration of Tregs to the tumor site, limiting the immunosuppressive microenvironment, and is associated with an increased presence of antitumoral CD8^+^ T cells and enhanced Granzyme B production. Ultimately, we demonstrate that dietary BRB-E is a viable chemopreventive strategy for at-risk populations in HNSCC. Identification of the specific BRB phytochemicals responsible for host T-cell immunomodulation is an important step in advancing this chemopreventive strategy against HNSCC.

## Data availability statement

The original contributions presented in the study are included in the article/supplementary material. Further inquiries can be directed to the corresponding author.

## Ethics statement

The animal study was reviewed and approved by Institutional Animal Care and Use Committee, The Ohio State University.

## Author contributions

NR and SO provided the overall study design. NR, FL, PU, MS, and AS conducted laboratory work and data acquisition. Data analysis and interpretation were carried out by NR, FL, PU, MS, AS, and SO. NR, FL, HP, AS, and SO contributed to the drafting, editing, and revision of the final manuscript. SO acquired the funding to conduct all experiments. All authors contributed to the article and approved the submitted version.

## Funding

This work was funded by the National Institutes of Health grant numbers K01CA207599 (NCI/NIH), R56DE030093 (NIDCR/NIH), and DP1DA054344 (NIDA/NIH), as well as the American Cancer Society (ACS), grant number RSG-19-079-01-TBG awarded to SO.

## Conflict of interest

The authors declare that the research was conducted in the absence of any commercial or financial relationships that could be construed as a potential conflict of interest.

## Publisher’s note

All claims expressed in this article are solely those of the authors and do not necessarily represent those of their affiliated organizations, or those of the publisher, the editors and the reviewers. Any product that may be evaluated in this article, or claim that may be made by its manufacturer, is not guaranteed or endorsed by the publisher.
